# Associations of oxidative balance score with hyperuricemia and gout among American adults: a population-based study

**DOI:** 10.3389/fendo.2024.1354704

**Published:** 2024-06-26

**Authors:** Kai Wang, Jinyi Wu, Minggang Deng, Jiaqi Nie, Fengxi Tao, Qingwen Li, Xin Luo, Fang Xia

**Affiliations:** ^1^ Department of Public Health, Wuhan Fourth Hospital, Wuhan, China; ^2^ Department of Psychiatry, Wuhan Mental Health Center, Wuhan, China; ^3^ Department of Psychiatry, Wuhan Hospital for Psychotherapy, Wuhan, China; ^4^ XiaoGan Center for Disease Control and Prevention, Xiaogan, China

**Keywords:** oxidative balance score, hyperuricemia, gout, oxidative stress, lifestyle

## Abstract

**Objective:**

The current study aimed to assess the relationships between oxidative balance score (OBS) and OBS subclasses (dietary and lifestyle OBS) with risks of hyperuricemia (HUA) and gout among American adults.

**Methods:**

Participants in the National Health and Nutrition Examination Survey from 2007 to 2018 were initially recruited and then the final sample was restricted to adults without missing values about serum uric acid, gout, OBS, and covariates. Rao-Scott adjusted chi-square test and analysis of variance were utilized to compare the baseline characteristics in adults of different quartiles of OBS, while the weighted stepped logistic regression models were used to explore the associations of overall, dietary, and lifestyle OBS with the risks of HUA and gout. Weighted restricted cubic spline analyses were conducted to explore the nonlinear dose-response associations.

**Results:**

The final sample consisted of 22,705 participants aged 20 years and older, which was representative of approximately 197.3 million non-institutionalized American adults. HUA and gout prevalence decreased with OBS quartiles. Compared with adults in the first quartile of OBS, those in the second (OR: 0.85, 95% CI: 0.72–0.99), third (OR: 0.71, 95% CI: 0.58–0.85), and fourth (OR: 0.48, 95% CI: 0.38–0.61) quartiles of OBS had reduced risks of hyperuricemia. Similarly, adults in the second (OR: 0.70, 95% CI: 0.51–0.97) quartile of OBS was associated with lower gout risk in comparison to adults in the lowest quartile. Regarding OBS subclasses, dietary and lifestyle OBS were both negatively correlated with the risk of HUA, and only higher lifestyle OBS was significantly associated with lower gout risk. Furthermore, the subgroup analyses and interaction effects also substantiated similar effects. Significant nonlinear dose-response relationships were observed between overall, dietary, and lifestyle OBS with HUA risk as well as that of lifestyle OBS with gout risk.

**Conclusion:**

This study strongly suggests the significant negative associations of OBS with HUA and gout in American adults and provides a dietary and lifestyle guideline to reduce the risks.

## 1 Introduction

Gout, characterized by the accumulation of monosodium urate crystals in joints and other structures, is the most common form of inflammatory arthritis, and the primary risk factor is the increased concentration of serum uric acid [hyperuricemia (HUA)] (1). The presence of gout and HUA is frequently associated with various cardiometabolic and renal comorbidities, resulting in a decline in quality of life and substantial treatment expenditures (2–4). It was reported that the prevalence of gout among American adults remained stable between 2007 and 2016, with 3.9%, 5.2%, and 2.7% among adults, male adults, and female adults in 2015–2016 (5). Moreover, HUA prevalence rates were 20.2% and 20.0% among American men and women, with the prevalence being stable between 2007 and 2016. A systematic analysis about healthy life expectancy for the Global Burden of Disease Study 2017 identified that gout contributed to 1.28 million disability-adjusted life years globally (6). Except for the overwhelming studies suggesting the elevated risks of cardiovascular disease (CVD) and chronic kidney disease (CKD) in patients with gout, several studies demonstrated the significant associations of gout with hypertension and diabetes (7–12).

Oxidative stress could result in a disruption of redox signaling pathways and relevant molecular damage, the definition of which is based on the imbalance between oxidants and antioxidants in favor of the oxidants (13). Oxidative balance score (OBS), firstly developed in 2002 and limited by previous literature about oxidants, only consisted of beta-carotene, vitamin C, and iron (14). With further explorations of studies about the antioxidant and oxidant properties in nutrients, lifestyle, and biomarkers, 21 different kinds of OBS were established and updated before 2019 (15). OBS in our study consisted of 16 dietary and 4 lifestyle antioxidants and oxidants to determine individual exposures to oxidants and antioxidants, with higher OBS indicating lower exposure to oxidants and higher exposure to antioxidants (16). In comparison with a single biomarker such as reactive oxygen species and thioredoxin-interacting protein, OBS is a quantified index that greatly improves the identification of individual oxidative stress level, with higher simplicity and comprehensibility (16). Furthermore, OBS has been proven to be linked to oxidative stress level in studies concerning adults in the United States, suggesting the validity of OBS in American adults (17, 18).

Uric acid is pro-oxidant, but it also has antioxidant properties, and the relationship between uric acid and oxidative stress is still under debate. The purine catabolism generates uric acid and product reactive oxygen species (ROS) to regulate the cell signaling pathways and cell redox state in which xanthine oxidoreductase is both involved, indicating the possible correlations of uric acid and oxidative stress (19, 20). Moreover, researchers found that the control of the inflammasome and pro-inflammatory cytokine release is strongly associated with the molecular mechanism under gouty inflammation. As a result, several emerging studies have proposed the novel hypothesis concerning uric acid and metabolic syndrome, in which increased oxidative stress was identified as a pathophysiological mechanism (21, 22). Urate was found to release free radicals and deactivate innate antioxidant enzymes to exacerbate oxidative stress (23). In addition, HUA can trigger inflammation during the pathogenesis of acute gout as nucleotide oligomerization domain-like receptor thermal protein domain-associated protein 3 (NLRP3) inflammasome and interleukin were activated (24, 25). Meanwhile, the inflammation and oxidative stress induced by HUA was found to be alleviated by the inhibition of NLRP3 inflammasome, which provides a potential therapeutic pathway to decrease uric acid and alleviate the syndrome of HUA (26). To our knowledge, no previous studies have assessed the associations of OBS and HUA with gout. Therefore, we aimed to explore the associations of OBS with HUA and gout in U.S. adults, as well as in various demographic subgroups with the National Health and Nutrition Examination Survey (NHANES).

## 2 Materials and methods

### 2.1 Study populations

NHANES is a population-based survey to assess health and nutrition status in the U.S. non-institutionalized populations, in which data were collected every 2 years as a cycle. Complex, multistage, probability sampling design, and oversampling of different subpopulations are adopted to make the participants in a 2-year survey representative for the national non-institutionalized population.

**Figure 1:**
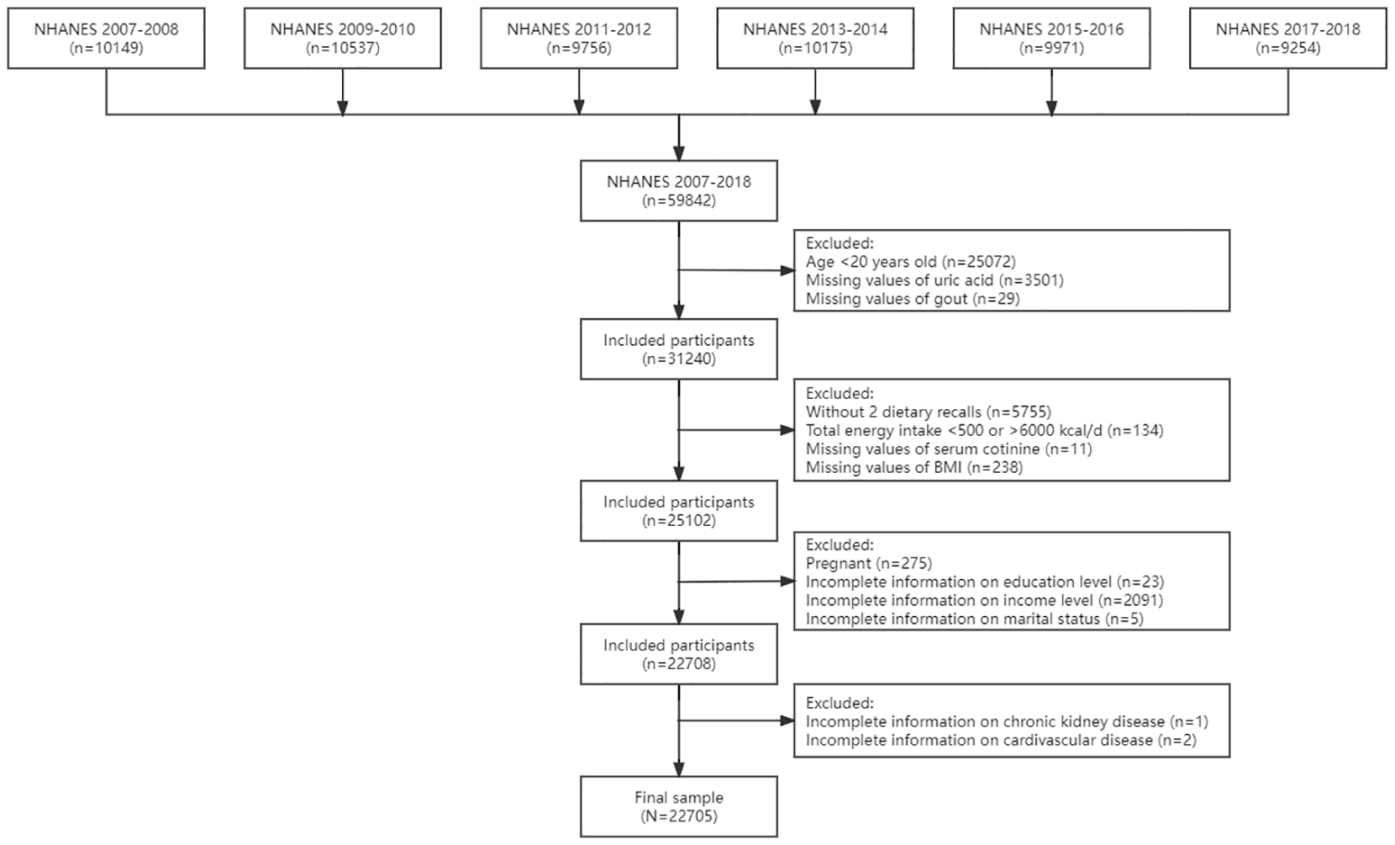
The flowchart of the sample design. NHANES, National Health and Nutrition Examination Survey.

In the current study, we included 31,240 adults aged 20 years and older without missing values of uric acid and self-reported gout in six cycles from NHANES 2007–2008 to 2017–2018. Detailed information of sampling and inclusion and exclusion criteria is shown in **Figure 1**. Adults without two dietary recalls (*n* = 5,755) and total energy intake >6,000 or <500 kcal per day (*n* = 134) were excluded. Furthermore, we excluded pregnant adults (*n* = 275) and adults without information on serum cotinine (*n* = 11), body mass index (BMI, *n* = 238), education (*n* = 23), family income (*n* = 2,091), and marital status (*n* = 5). After excluding participants without detailed information on CVD (*n* = 2) and CKD (*n* = 1), the final sample included 22,705 adults.

### 2.2 Exposures

OBS in the current study was established by Zhang et al. and has been validated by multiple studies (see **Supplementary Table S1**) (16). OBS subclasses included dietary and lifestyle OBS, and the latter consisted of BMI, physical activity, alcohol consumption, and smoking status (measured by serum cotinine) while the former was composed of 16 dietary nutrients intake such as fiber and carotene. In addition, 20 components were further classified into pro-oxidants (total fat, iron, alcohol consumption, BMI, and smoking status) and antioxidants (the other 15 components) by the oxidative properties. Dietary nutrients and alcohol intakes were assessed using the mean of two 24-h dietary recalls. BMI was calculated as weight (kilograms) divided by height (meter) squared, and smoking status was assessed by cotinine but not nicotine since the half-life of cotinine in plasma is substantially longer than that of nicotine. Physical activity was determined by leisure-time physical activity (LTPA), which is calculated as the duration of moderate physical activity plus double the duration of vigorous physical activity according to the physical activity guidelines for Americans (27). Since the tertiles of OBS components varied greatly in sex, OBS components were stratified to sex and scored from 0 to 2 on the basis of tertiles. The highest tertile of oxidants was scored 0 and the lowest tertile was scored 2 while the scoring method of pro-oxidants was just the opposite, suggesting that OBS is in favor of antioxidants and lower OBS indicates higher exposure to oxidants. For the convenience of further comparisons, we categorized OBS into quartiles, and set the lowest quartile as the reference group for subsequent weighted stepped logistic regression models.

### 2.3 Assessment of covariates

Similar to our previous studies, covariates of interest consisted of demographic variable, dietary confounding factors, and comorbidities. Demographic variables were composed of sex (male and female), age group (20–39 years, 40–59 years, and 60–80 years), race (non-Hispanic White, non-Hispanic Black, Mexican Americans, and other races), education level (less than high school degree, high school graduate or general educational development, and some college or above), the ratio of family income to poverty (PIR) (the measurement of family income level: low, PIR ≤ 1.3; medium, 1.3 < PIR < 3.5; high, PIR ≥ 3.5), and marital status (married or living with partner; divorced, separated, or widowed; and never married). Dietary confounding factors consisted of healthy eating index-2015 (HEI-2015) and total energy intake (expressed as kilocalories per day) to eliminate the effect of energy intake and diet quality, which, as assessed by HEI-2015, has been found to be negatively associated with the risks of HUA and gout in our previous study (28). Comorbidities included multiple chronic non-communicable diseases such as hypertension, CVD, diabetes, and CKD on account of the positive associations with HUA and gout. The diagnostic criteria of comorbidities were based on blood pressure measurements, biochemical indicators, and self-report by a professional doctor, and detailed information could be obtained in our previous studies (29–32).

### 2.4 Outcome ascertainment

The serum uric acid concentration was measured using a timed endpoint method, where uric acid was oxidized by uricase to produce allantoin and hydrogen peroxide. The calculation of uric acid was based on the absorbance, the change of which is directly proportional to the concentration of serum uric acid. The laboratory procedure manual of standard biochemistry profile presented detailed information about the test principle and clinical relevance. HUA was defined as the serum concentration of uric acid >7.0 and >5.7 mg/dL in male and female adults, while gout was defined as self-report of being diagnosed by a professional doctor in medical condition questionnaires (28, 33).

### 2.5 Statistical analysis

Proper weights (dietary 2-day sample weight, WTDR2D), clustering, and stratification were considered according to analytic guidelines released by the Centers for Disease Control and Prevention. Moreover, the final sample in our study consisted of six consecutive cycles; thus, we utilized WTDR2D divided by 6 as the final weight to make it representative of the national non-institutionalized population (34).

Statistical descriptions involved presenting continuous variables as weighted means (standard deviations) and categorical variables as numbers (weighted percentages). To compare the characteristics between adults of different OBS quartile groups, Rao-Scott-adjusted chi-square tests and analyses of variance were used. Weighted logistic regression models, both univariate and multivariate, were utilized to explore the relationship between OBS and HUA with gout in the general population. Model 1 was adjusted for demographic variables, and model 2 was additionally adjusted for dietary confounding factors while model 3 as the fully adjusted model was additionally adjusted for comorbidities based on model 2. Moreover, weighted stepped logistic regression models of gout were additionally adjusted for HUA. OBS subclasses as dietary and lifestyle OBS were separately analyzed to explore the associations with HUA and gout. Furthermore, trend tests (*p* for trend) were conducted by treating the OBS quartiles as a continuous variable and re-running the corresponding logistic regression models. Stratified analyses were performed to investigate whether the associations varied by demographic variables and interaction effects were tested with likelihood tests. Weighted restricted cubic splines (RCS) were employed to assess the nonlinear and dose–response correlations of overall, dietary, and lifestyle OBS with HUA and gout.

R software (version 4.2.2) was used for analyses of variance and all other statistical analyses were conducted in Stata software (version 17.0, StataCorp LLC). All statistical tests were two-sided, and a significance level of α = 0.05 was considered.

## 3 Results

### 3.1 Characteristics

Characteristics in adults of different OBS quartiles are shown in **Table 1**. The final sample included 22,705 American adults representative for 197.3 million non-institutionalized U.S. adults [mean (SD) OBS, 20.42 (7.27); 10,964 (weighted 48.4%) men; 10,195 (weighted 68.3%) non-Hispanic White; 4,918 (weighted 20.6%) HUA; 1,102 (weighted 4.1%) gout]. In comparison with adults in the lowest quartile of OBS, those in higher quartiles of OBS were more likely to be young, non-Hispanic White, and married or living with partner. Meanwhile, adults in higher quartiles of OBS were less likely to be comorbid with hypertension, CVD, diabetes, CKD, HUA, and gout. Moreover, adults in higher quartiles of OBS had a higher education level, income level, total energy intake, and HEI-2015 scores. Nevertheless, no significant differences of OBS were found between male and female adults.

**Table 1:** The characteristics by quartiles of the OBS.

Characteristics	Overall (*N* = 22,705)	Q1 (3–14) (*n* = 6,577)	Q2 (15–20) (*n* = 5,833)	Q3 (21–26) (*n* = 5,647)	Q4 (27–37) (*n* = 4,648)	*p*-value
**Sex** (*n*/%)						0.37
Male	10,964 (48.4)	3,246 (48.6)	2,865 (49.4)	2,708 (48.6)	2,145 (46.9)	
Female	11,741 (51.6)	3,331 (51.4)	2,968 (50.6)	2,939 (51.4)	2,503 (53.1)	
**Age group** (*n*/%)						<0.001
Young adults (20–39 years)	7,257 (36.1)	1,904 (34.8)	1,831 (34.9)	1,858 (36.5)	1,664 (38.2)	
Middle-aged adults (40–59 years)	7,679 (37.7)	2,116 (37.0)	1,905 (35.8)	1,995 (38.5)	1,663 (39.4)	
Older adults (60–80 years)	7,769 (26.3)	2,557 (28.3)	2,097 (29.4)	1,794 (24.9)	1,321 (22.4)	
**Race** (*n*/%)						<0.001
Non-Hispanic White	10,195 (68.3)	2,702 (62.6)	2,629 (68.0)	2,583 (69.4)	2,281 (73.1)	
Non-Hispanic Black	4,597 (10.3)	1,940 (17.3)	1,138 (10.2)	925 (8.2)	594 (5.5)	
Mexican Americans	3,232 (8.4)	798 (7.9)	862 (8.8)	870 (8.7)	702 (8.1)	
Other races	4,681 (13.1)	1,137 (12.2)	1,204 (13.0)	1,269 (13.7)	1,071 (13.3)	
**Education level** (*n*/%)						<0.001
<High school	4,960 (14.1)	1,931 (21.6)	1,370 (15.3)	1,022 (11.5)	637 (8.1)	
High school	5,181 (22.8)	1,825 (29.5)	1,360 (25.5)	1,212 (21.1)	784 (15.1)	
>High school	12,564 (63.1)	2,821 (48.9)	3,103 (59.3)	3,413 (67.4)	3,227 (76.7)	
**Family income level** (*n*/%)						<0.001
Low	7,052 (21.6)	2,637 (31.5)	1,820 (21.7)	1,511 (18.0)	1,084 (15.4)	
Medium	8,558 (34.8)	2,580 (38.3)	2,273 (36.5)	2,097 (34.4)	1,608 (29.8)	
High	7,095 (43.6)	1,360 (30.2)	1,740 (41.8)	2,039 (47.6)	1,956 (54.9)	
**Marital status** (*n*/%)						<0.001
Married or living with partner	13,699 (63.3)	3,609 (56.4)	3,527 (63.8)	3,571 (65.8)	2,992 (67.2)	
Divorced, separated, or widowed	5,040 (18.2)	1,733 (22.7)	1,340 (19.2)	1,147 (16.3)	820 (14.6)	
Never married	3,966 (18.5)	1,235 (20.1)	966 (17.0)	929 (17.9)	836 (18.2)	
**Hypertension** (*n*/%)	10,936 (41.8)	3,628 (47.8)	2,938 (45.0)	2,557 (40.0)	1,813 (34.2)	<0.001
**CVD** (*n*/%)	2,513 (8.6)	1,035 (12.3)	640 (8.6)	518 (8.0)	320 (5.3)	<0.001
**Diabetes** (*n*/%)	4,201 (13.5)	1,550 (17.8)	1,138 (13.7)	948 (13.3)	565 (9.0)	<0.001
**CKD** (*n*/%)	12,266 (27.4)	3,661 (28.6)	3,235 (29.6)	2,960 (25.9)	2,410 (25.7)	0.01
**HUA** (*n*/%)	4,918 (20.6)	1,731 (25.2)	1,359 (22.7)	1,085 (19.6)	743 (14.6)	<0.001
**Gout** (*n*/%)	1,102 (4.1)	427 (5.6)	266 (3.9)	257 (4.1)	152 (2.9)	<0.001
**Total energy intake (kcal), mean (SD)**	2,095.51 (785.63)	1,545.75 (523.51)	1,944.25 (602.80)	2,252.45 (685.97)	2,648.26 (855.23)	<0.001
**HEI-2015 score, mean (SD)**	53.87 (13.41)	46.78 (11.45)	51.76(12.33)	56.03(12.81)	61.03(12.81)	<0.001

CKD, chronic kidney disease; CVD, cardiovascular disease; HEI-2015, healthy eating index-2015; HUA, hyperuricemia; OBS, oxidative balance score.

### 3.2 Associations between overall OBS with HUA and gout

Weighted stepped logistic regression models in **Table 2** revealed that higher OBS quartiles were associated with lower risks of HUA and gout. Specifically, the second (OR: 0.85, 95% CI: 0.72–0.99), the third (OR: 0.71, 95% CI: 0.58–0.85), and the highest quartiles (OR: 0.48, 95% CI: 0.38–0.61) of OBS were associated with lower risks of HUA in model 3 when compared with the lowest quartile. Furthermore, adults in the second quartile of OBS had 30% (OR: 0.70, 95% CI: 0.51–0.97) reduced risk of gout, but adults in the third and highest quartiles did not show significant lower risks in comparison with the lowest quartile.

**Table 2:** The relationship between OBS and HUA with gout.

OBS	HUA			Gout		
	Model 1	Model 2	Model 3	Model 1	Model 2	Model 3
Q1	Ref	Ref	Ref	Ref	Ref	Ref
Q2	0.88 (0.77,1.01)	0.84 (0.72,0.98)	0.85 (0.72,0.99)	0.69 (0.50,0.92)	0.66 (0.48,0.91)	0.70 (0.51,0.97)
Q3	0.75 (0.64,0.87)	0.69 (0.57,0.83)	0.71 (0.58,0.85)	0.82 (0.61,1.10)	0.73 (0.53,1.01)	0.81 (0.59,1.10)
Q4	0.53 (0.45,0.62)	0.46 (0.37,0.58)	0.48 (0.38,0.61)	0.67 (0.50,0.92)	0.54 (0.35,0.82)	0.68 (0.45,1.04)
*p* for trend	<0.001	<0.001	<0.001	0.04	0.06	0.12

HUA, hyperuricemia; OBS, oxidative balance score.

Model 1 was adjusted for demographic data (sex, age group, education level, income level, and marital status).

Model 2 was adjusted for demographic data, total energy intake, and HEI-2015.

Model 3 was adjusted for demographic data, total energy intake, HEI-2015, and disease conditions (hypertension, CVD, diabetes, and CKD).

### 3.3 Associations of dietary and lifestyle OBS with HUA and gout

To assess the independent effect of OBS subclasses, dietary and lifestyle OBS were included separately in the weighted stepped logistic regression models and the results are shown in **Table 3**. After multivariate adjustments, adults in the highest quartile of dietary OBS had 29% (OR: 0.71, 95% CI: 0.57–0.89) reduced HUA risk in comparison with the lowest quartile. Moreover, adults in the second, third, and highest quartiles of lifestyle OBS had 29%, 45%, and 65% lower risk of HUA, and the OR and 95% CI were 0.71 (0.62–0.80), 0.55 (0.48–0.64), and 0.35 (0.30–0.42), respectively. Gout risks did not differ by quartiles of dietary OBS in all models, but adults in the second, third, and highest quartiles of lifestyle OBS had 28%, 35%, and 45% reduced gout risk in model 3, and the OR and 95% CI were 0.72 (0.55–0.94), 0.65 (0.45–0.94), and 0.55 (0.41–0.75), respectively.

**Table 3:** The relationship between dietary OBS and lifestyle OBS with HUA and gout.

OBS	HUA			Gout		
	Model 1	Model 2	Model 3	Model 1	Model 2	Model 3
Dietary OBS						
Q1 (1–10)	Ref	Ref	Ref	Ref	Ref	Ref
Q2 (11–16)	0.95 (0.83,1.09)	0.97 (0.83,1.13)	0.96 (0.83,1.13)	0.81 (0.62,1.05)	0.83 (0.62,1.12)	0.84 (0.62,1.13)
Q3 (17–21)	0.85 (0.73,0.98)	0.87 (0.73,1.05)	0.89 (0.73,1.07)	1.04 (0.75,1.44)	1.09 (0.75,1.57)	1.15 (0.80,1.66)
Q4 (22–32)	0.68 (0.58,0.79)	0.71 (0.57,0.88)	0.71 (0.57,0.89)	0.69 (0.52,0.91)	0.73 (0.50,1.08)	0.81 (0.55,1.19)
*p* for trend	<0.001	0.01	0.01	0.31	0.01	0.76
Lifestyle OBS						
Q1 (0–3)	Ref	Ref	Ref	Ref	Ref	Ref
Q2 (4)	0.70 (0.61,0.79)	0.69 (0.61,0.79)	0.71 (0.62,0.80)	0.66 (0.51,0.86)	0.86 (0.51,0.86)	0.72 (0.55,0.94)
Q3 (5)	0.54 (0.47,0.62)	0.53 (0.46,0.62)	0.55 (0.48,0.64)	0.53 (0.38,0.76)	0.54 (0.38,0.77)	0.65 (0.45,0.94)
Q4 (6–8)	0.33 (0.29,0.39)	0.33 (0.28,0.39)	0.35 (0.30,0.42)	0.39 (0.28,0.54)	0.40 (0.29,0.55)	0.55 (0.41,0.75)
*p* for trend	<0.001	<0.001	<0.001	<0.001	<0.001	<0.001

HUA, hyperuricemia; OBS, oxidative balance score.

Model 1 was adjusted for demographic data (sex, age group, education level, income level, and marital status).

Model 2 was adjusted for demographic data, total energy intake, and HEI-2015.

Model 3 was adjusted for demographic data, total energy intake, HEI-2015, and disease conditions (hypertension, CVD, diabetes, and CKD).

### 3.4 Subgroup analyses and interaction effects of the relationships between OBS and HUA according to demographic variables

**Table 4** reveals the associations between OBS and HUA in demographic subpopulations. The significant negative associations of OBS quartile and the risk of HUA were identified in various subpopulations except for Mexican Americans and divorced, separated, or widowed adults. In addition, a significant interaction effect of education level and OBS on the HUA risk was discovered (*p* = 0.01), and no other interaction was inferred, shown as higher education in the same quartile of OBS being correlated with reduced ORs. In other words, the protective effect of OBS against HUA risk was the best among adults with higher education level.

**Table 4:** The relationship between OBS and HUA in demographic subgroups.

Characteristics	Q1	Q2	Q3	Q4	*p* for trend	*p* for interaction
**Sex**						0.48
Male	Ref	0.75 (0.60,0.95)	0.62 (0.48,0.80)	0.41 (0.31,0.56)	<0.001	
Female	Ref	0.94 (0.77,1.16)	0.80 (0.62,1.04)	0.56 (0.41,0.77)	<0.001	
**Age group**						0.30
Young adults (20–39 years)	Ref	0.67 (0.52,0.85)	0.50 (0.37,0.67)	0.30 (0.21,0.43)	<0.001	
Middle-aged adults (40–59 years)	Ref	0.97 (0.72,1.31)	0.86 (0.62,1.18)	0.58 (0.38,0.90)	0.01	
Older adults (60–80 years)	Ref	0.93 (0.75,1.14)	0.83 (0.65,1.06)	0.67 (0.46,0.97)	0.03	
**Race**						0.47
Non-Hispanic White	Ref	0.84 (0.68,1.05)	0.71 (0.55,0.90)	0.44 (0.33,0.60)	<0.001	
Non-Hispanic Black	Ref	1.00 (0.77,1.29)	0.81 (0.59,1.11)	0.66 (0.45,0.96)	0.03	
Mexican Americans	Ref	1.09 (0.64,1.85)	0.89 (0.47,1.70)	0.88 (0.43,1.81)	0.62	
Other races	Ref	0.77 (0.54,1.09)	0.65 (0.45,0.95)	0.48 (0.31,0.75)	0.01	
**Education level**						0.01
<High school	Ref	0.82 (0.60,1.11)	0.86 (0.59,1.25)	0.47 (0.29,0.78)	0.02	
High school	Ref	0.73 (0.54,0.99)	0.82 (0.60,1.13)	0.63 (0.40,0.99)	0.06	
>High school	Ref	0.90 (0.73,1.11)	0.64 (0.50,0.82)	0.44 (0.33,0.61)	<0.001	
**Income level**						0.07
Low	Ref	0.70 (0.55,0.88)	0.71 (0.52,0.98)	0.57 (0.40,0.80)	0.01	
Medium	Ref	0.79 (0.62,1.01)	0.74 (0.55,1.01)	0.50 (0.35,0.72)	0.01	
High	Ref	1.03 (0.78,1.34)	0.71 (0.52,0.96)	0.46 (0.31,0.69)	<0.001	
**Marital status**						0.54
Married or living with partner	Ref	0.89 (0.73,1.08)	0.68 (0.53,0.87)	0.43 (0.32,0.58)	<0.001	
Divorced, separated, or widowed	Ref	0.93 (0.70,1.25)	0.98 (0.72,1.34)	0.78 (0.53,1.16)	0.32	
Never married	Ref	0.65 (0.46,0.92)	0.56 (0.38,0.82)	0.42 (0.25,0.71)	0.01	

The fully adjusted model was adjusted for demographic data, total energy intake, HEI-2015, and disease conditions (hypertension, CVD, diabetes, and CKD).

HUA, hyperuricemia; OBS, oxidative balance score.

Since only higher lifestyle but not dietary OBS quartiles were found to be correlated with lower gout risks, we performed the subgroup analyses and tested the interaction effect on the associations of lifestyle OBS with gout in **Table 5**. Except for young adults, Mexican Americans, low and medium family income level, and divorced, separated, or widowed adults, the negative relationships between lifestyle OBS and gout risk were significant in various subgroups. Moreover, no significant interaction effects were observed, indicating that the correlations between lifestyle OBS and gout risks did not differ by demographic variables.

**Table 5:** The relationship between lifestyle OBS and gout in demographic subgroups.

Characteristics	Q1	Q2	Q3	Q4	*p* for trend	*p* for interaction
**Sex**						0.74
Male	Ref	0.67 (0.48,0.93)	0.65 (0.41,1.02)	0.57 (0.39,0.82)	0.01	
Female	Ref	0.85 (0.55,1.32)	0.66 (0.38,1.16)	0.55 (0.30,0.99)	0.02	
**Age group**						0.97
Young adults (20–39 years)	Ref	0.93 (0.30,2.91)	0.68 (0.18,2.52)	0.81 (0.28,2.30)	0.57	
Middle-aged adults (40–59 years)	Ref	0.70 (0.42,1.14)	0.71 (0.40,1.25)	0.51 (0.27,0.98)	0.02	
Older adults (60–80 years)	Ref	0.81 (0.59,1.10)	0.68 (0.45,1.03)	0.62 (0.43,0.88)	0.01	
**Race**						0.48
Non-Hispanic White	Ref	0.77 (0.56,1.07)	0.72 (0.46,1.14)	0.53 (0.36,0.77)	0.01	
Non-Hispanic Black	Ref	0.60 (0.40,0.91)	0.47 (0.27,0.83)	0.42 (0.24,0.72)	0.01	
Mexican Americans	Ref	0.56 (0.28,1.13)	0.52 (0.25,1.07)	0.44 (0.10,1.86)	0.16	
Other races	Ref	0.60 (0.31,1.17)	0.49 (0.24,0.98)	0.88 (0.42,1.83)	0.65	
**Education level**						0.64
<High school	Ref	0.55 (0.36,0.84)	0.90 (0.38,2.10)	0.47 (0.26,0.86)	0.13	
High school	Ref	0.44 (0.25,0.77)	0.44 (0.25,0.77)	0.41 (0.21,0.80)	0.01	
>High school	Ref	0.79 (0.54,1.14)	0.70 (0.42,1.17)	0.62 (0.42,0.93)	0.03	
**Income level**						0.86
Low	Ref	0.71 (0.48,1.05)	0.64 (0.35,1.17)	0.52 (0.26,1.04)	0.02	
Medium	Ref	0.92 (0.57,1.49)	0.73 (0.43,1.24)	0.70 (0.43,1.14)	0.09	
High	Ref	0.57 (0.35,0.92)	0.62 (0.37,1.06)	0.51 (0.31,0.84)	0.02	
**Marital status**						0.51
Married or living with partner	Ref	0.71 (0.49,1.02)	0.64 (0.42,0.98)	0.51 (0.35,0.76)	0.01	
Divorced, separated, or widowed	Ref	0.84 (0.53,1.34)	0.83 (0.46,1.48)	0.96 (0.49,1.87)	0.74	
Never married	Ref	0.71 (0.29,1.75)	0.28 (0.11,0.72)	0.44 (0.13,1.42)	0.04	

The fully adjusted model was adjusted for demographic data, total energy intake, HEI-2015 and disease conditions (hypertension, CVD, diabetes and CKD).

HUA, hyperuricemia; OBS, oxidative balance score.

### 3.5 Nonlinear dose–response relationships of OBS, dietary OBS, and lifestyle OBS with HUA and gout

**Figure 2:**
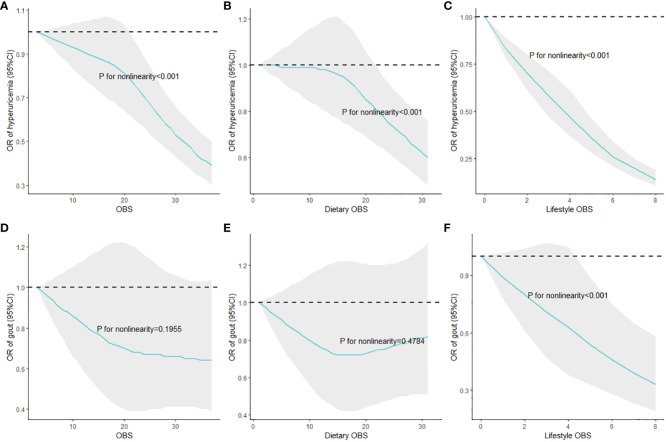
Nonlinear associations of overall, dietary, and lifestyle OBS with HUA and gout risks. **(A)** OBS and hyperuricemia; **(B)** dietary OBS and hyperuricemia; **(C)** lifestyle OBS and hyperuricemia; **(D)** OBS and gout; **(E)** dietary OBS and gout; **(F)** lifestyle OBS and gout. Multivariate adjustments included demographic variables, total energy intake per day, HEI-2015, and comorbidities; HUA, hyperuricemia; OBS, oxidative balance score; RCS, restricted cubic splines.

Weighted RCS were employed to assess the nonlinear dose–response relationships of overall, dietary, and lifestyle OBS with the risks of HUA and gout, and the results are shown in **Figure 2**. **Figures 2A–C** show the significant nonlinear correlations of overall, dietary, and lifestyle OBS with HUA risks, in which higher OBS indicated lower ORs. In addition, **Figures 2D–F** show the associations of overall, dietary, and lifestyle OBS with gout risks. Specifically, only the significant nonlinear association of lifestyle OBS with gout risk was observed, and higher lifestyle OBS suggested reduced ORs of gout when it was relatively high.

## 4 Discussion

Through our final sample from NHANES 2007–2018 and multiple statistical methods, we found the strong negative associations of overall, dietary, and lifestyle OBS with HUA risk. Nevertheless, no significant reduced gout risk of higher dietary OBS quartiles were shown while higher overall OBS and lifestyle OBS were correlated with decreased gout risk. Moreover, the relationship between OBS and HUA risk and the association of lifestyle OBS with gout risk were stable in various demographic subgroups, suggesting the robustness of our results. Furthermore, weighted RCS indicated the nonlinear association of overall, dietary, and lifestyle OBS with HUA risk, as well as the correlation between lifestyle OBS and gout risk.

OBS in the current study, unlike other indices that consisted of antioxidant or pro-oxidant biomarkers, was composed of 16 dietary and 4 lifestyle factors and was utilized to reflect the individual oxidative stress level. Compared with other kinds of OBS including biochemical indicators, the current OBS is not only an index but also a dietary and lifestyle guideline, in which antioxidant dietary factors and lifestyles such as dietary fiber intake and physical activity are encouraged while pro-oxidant dietary factors and lifestyles like dietary total fat and alcohol intake are discouraged. OBS was established to reflect the oxidative stress and explore the associations of oxidative stress and leukocyte telomere length, and the results revealed the negative relationships in female adults, consistent with previous experiments and reviews (35–38). Furthermore, two epidemiological studies concerning NHANES databases assessed the relationships between OBS and cognitive function with depression symptoms in which oxidative stress serves a mediation role, indirectly demonstrating the significant associations between OBS and oxidative stress (17, 18).

Multiple studies have assessed the association between uric acid and oxidative stress and found the dual action of uric acid, specifically manifesting as having both oxidant and antioxidant properties. It has been found that high levels of serum uric acid may contribute to the pro-oxidative and pro-inflammatory state (22). Furthermore, the correlation of oxidative stress with inflammation is bi-directional, in which the NLRP3 inflammasome could be activated by the accumulation of mitochondria that produce ROS, and high uric acid increased oxidative stress measured by thioredoxin-interacting protein that acts by inhibiting thioredoxin activity (38–40). Furthermore, oxidative stress and inflammation were both involved in the typical impairments resulting from asymptomatic HUA, and our results on the correlation between OBS and HUA risk confirm the potential oxidative stress and inflammation mechanism (28). For example, uric acid was thought to increase the production of ROS in mitochondrion and then lead to higher oxidative stress level, while HUA activated NLRP3 to trigger oxidative stress during the pathogenesis of acute gout (41, 42). Xanthine oxidase, the enzyme that catalyzes the formation of uric acid, was thought to help in inhibiting the adverse effect of ROS by providing a crucial source of nitric oxide, suggesting the negative association between uric acid and oxidative stress (43, 44). Even so, more studies concluded that uric acid contributed to an elevated oxidative stress level regardless of xanthine oxidoreductase activity and was thought to induce oxidative stress (45, 46). To sum up, our results confirm the significant negative associations of OBS with HUA and gout risks, indicating the significant positive associations of oxidative stress with HUA and gout risks, which is acknowledged by the scientific community.

Further analyses of OBS subclasses demonstrated the significant negative associations of dietary and lifestyle OBS with HUA. Multiple dietary nutrient intake including B group vitamins, such as vitamin B6, vitamin B12, and folate, and other nutrients such as fiber, retinol, zinc, magnesium, vitamin C, and vitamin E has been proven to be negatively correlated with HUA risk, while dietary fat intake is suggested to be positively related to HUA risk in men with CKD (47–54). Nevertheless, dietary OBS was significantly associated with HUA but not gout, and more high-quality studies such as randomized controlled trials and prospective cohort studies are urged to verify the validity of our results and explore possible mechanisms. After consulting the relevant literature, we found that most studies focus on specific foods, diet patterns, and diet quality, but the significant association of nutrient intake with gout risk is rarely reported, indicating that the existence of a potential molecular mechanism of nutrient intake in the progression from HUA to gout leads to the significant association of dietary OBS with HUA but not gout. Lifestyle OBS is a factor related to HUA and gout, in which cohort studies and a randomized controlled trial have suggested the protective role of physical activity (55–57). Alcohol consumption and smoking have been recognized as significant risk factors for HUA and gout, and greater BMI increases the risk of HUA and gout (58–62).

The results of subgroup analyses, interaction effects, and weighted RCS and WQS regression models revealed the robust and negative associations of OBS with HUA risk as well as that of lifestyle OBS with gout risk. Moreover, adults with more than high school degree had the lowest HUA risk, which is different from our previous studies (28). As shown in weighted RCS models, nonlinear correlation persisted between overall, dietary, and lifestyle OBS with HUA risk, indicating that HUA risk did not decrease with OBS in a linear manner. Taking the slope into account, the dose–response curve was steeper between lifestyle OBS and HUA risk than that between OBS and dietary OBS and HUA risk, and the protective role of OBS and dietary OBS at a relatively low level against HUA did not exist in comparison with the lowest score. Furthermore, only a nonlinear association of lifestyle OBS with gout risk was observed. Specifically, the dose–response curves were steeper when OBS and dietary OBS were relatively high, indicating that small efforts of adults with relatively higher OBS and dietary OBS could bring huge reduced risks of HUA. Meanwhile, the dose–response curves of OBS and dietary OBS with HUA risk were moderate and not significant, encouraging adults to obtain higher OBS and dietary OBS score to reduce HUA risks. Furthermore, we conducted five sensitivity analyses to test the stability of our results, which are shown in **Supplementary Table S2**.

The major advantage of the current study is that the final sample was derived from a nationally representative large-scale survey and the combination of data in six cycles, which increased the sample size and expanded the generalizability of our findings. Furthermore, the employment of subgroup analyses and interaction effects identified the associations in subpopulations and enhanced the robustness. Finally, weighted RCS models illustrated the comprehensive and intuitive nonlinear dose–response curves. Nevertheless, several limitations in our study must be pointed out. Firstly, no causalities but only associations were determined in this study since the original data were from the cross-sectional study. Secondly, more prospective and high-quality studies are essential to evaluate the effectiveness of OBS on oxidative stress. Subsequently, gout and some covariates were based on self-report but not medical records or medication, diminishing the credibility of relevant information. Finally, quartiles of OBS were generated and utilized in the statistical analyses, weakening the comparability to other specific populations.

## Data availability statement

The datasets presented in this article are not readily available because it could be obtained in NHANES. Requests to access the datasets should be directed to https://www.cdc.gov/nchs/nhanes/ContinuousNhanes/Default.aspx?BeginYear=%202005.

## Author contributions

KW: Data curation, Formal analysis, Methodology, Software, Supervision, Validation, Visualization, Writing – original draft. JW: Methodology, Writing – review & editing. MD: Data curation, Formal analysis, Writing – review & editing. JN: Data curation, Formal analysis, Writing – review & editing. FT: Writing – review & editing. QL: Writing – review & editing. XL: Writing – review & editing. FX: Writing – review & editing.
