# Supplementary material for: Associations of oxidative balance score with hyperuricemia and gout among American adults: a population-based study
Source: Front Endocrinol (Lausanne). 2024 Jun 26;15:1354704. doi: 10.3389/fendo.2024.1354704 (PMC11233537; doi:10.3389/fendo.2024.1354704)
Supplement: Supplementary file 1 [file Table_1.docx]

**Table S1** Components of the oxidative balance score

| OBS components | Property | Male | | | Female | | |
| --- | --- | --- | --- | --- | --- | --- | --- |
|  |  | 0 | 1 | 2 | 0 | 1 | 2 |
| Dietary OBS components | | | | | | | |
| Dietary fiber (g/d) | A | <13.80 | 13.80-21.30 | ≥21.30 | <11.50 | 11.50-17.45 | ≥17.45 |
| Carotene (RE/d) | A | <159.25 | 159.25-658.75 | ≥658.75 | <175.83 | 175.83-721.33 | ≥721.33 |
| Riboflavin (mg/d) | A | <1.88 | 1.88-2.70 | ≥2.70 | <1.44 | 1.44-2.07 | ≥2.07 |
| Niacin (mg/d) | A | <24.10 | 24.10-33.62 | ≥33.62 | <16.80 | 16.80-23.63 | ≥23.63 |
| Vitamin B_6_ (mg/d) | A | <1.82 | 1.82-2.68 | ≥2.68 | <1.33 | 1.33-1.94 | ≥1.94 |
| Total folate (mcg/d) | A | <339.50 | 339.50-506.00 | ≥506.00 | <263.00 | 263.00-388.50 | ≥388.50 |
| Vitamin B_12_ (mcg/d) | A | <3.76 | 3.76-6.38 | ≥6.38 | <2.71 | 2.61-4.61 | ≥4.61 |
| Vitamin C (mg/d) | A | <39.65 | 39.65-97.85 | ≥97.85 | <38.60 | 38.60-87.65 | ≥87.65 |
| Vitamin E (ATE) (mg/d) | A | <6.58 | 6.58-10.56 | ≥10.56 | <5.42 | 5.42-8.70 | ≥8.70 |
| Calcium (mg/d) | A | <793.00 | 793.00-1213.00 | ≥1213.00 | <647.00 | 647.00-967.00 | ≥967.00 |
| Magnesium (mg/d) | A | <270.00 | 270.00-375.50 | ≥375.50 | <214.50 | 214.50-297.00 | ≥297.00 |
| Zinc (mg/d) | A | <10.21 | 10.21-14.79 | ≥14.79 | <7.42 | 7.42-10.61 | ≥10.61 |
| Copper (mg/d) | A | <1.08 | 1.08-1.53 | ≥1.53 | <0.88 | 0.85-1.24 | ≥1.24 |
| Selenium (mcg/d) | A | <106.50 | 106.50-147.80 | ≥147.80 | <76.30 | 76.30-107.20 | ≥107.20 |
| Total fat (g/d) | P | ≥105.18 | 73.34-105.18 | <73.34 | ≥77.57 | 54.22-77.57 | <54.22 |
| Iron (mg/d) | P | ≥18.66 | 12.87-18.66 | <12.87 | ≥14.16 | 9.86-14.16 | <9.86 |
| Lifestyle OBS components | | | | |  |  |  |
| Physical activity | A | 0 | 0-240 | ≥240 | 0 | 0-175 | ≥175 |
| Alcohol intake | P | ≥30 | 0-30 | 0 | ≥15 | 0-15 | 0 |
| Body mass index (kg/m^2^) | P | ≥30.60 | 26.09-30.60 | <26.09 | ≥31.30 | 25.03-31.30 | <25.03 |
| Cotinine (ng/mL) | P | ≥0.53 | 0.018-0.53 | <0.018 | ≥0.082 | 0.011-0.082 | <0.011 |

A, antioxidant; ATE, alpha-tocopherol equivalent; OBS, oxidative balance score; P, pro-oxidant; RE, retinol equivalent.

**Table S2** The sensitivity analyses of the associations of the OBS with HUA and gout

| OBS | HUA | | | Gout | | |
| --- | --- | --- | --- | --- | --- | --- |
|  | Model 1 | Model 2 | Model 3 | Model 1 | Model 2 | Model 3 |
| Sensitivity analysis 1 (HEI-2015 not adjusted for) | | | | | | |
| Q1 | ref | ref | ref | ref | ref | ref |
| Q2 | 0.88(0.77,1.01) | 0.84(0.73,0.98) | 0.86 (0.74,1.01) | 0.69(0.50,0.92) | 0.66(0.49,0.90) | 0.69 (0.50,0.93) |
| Q3 | 0.75(0.64,0.87) | 0.70(0.59,0.82) | 0.73 (0.61,0.86) | 0.82(0.61,1.10) | 0.74(0.56,0.97) | 0.77 (0.59,1.01) |
| Q4 | 0.53(0.45,0.62) | 0.47(0.39,0.57) | 0.50 (0.41,0.62) | 0.67(0.50,0.92) | 0.57(0.40,0.80) | 0.63 (0.45,0.89) |
| P for trend | <0.001 | <0.001 | <0.001 | 0.04 | 0.003 | 0.015 |
| Sensitivity analysis 2 (HEI-2015 excluding the components of saturated fats and fatty acids) | | | | | | |
| Q1 | ref | ref | ref | ref | ref | ref |
| Q2 | 0.88(0.77,1.01) | 0.86(0.74,1.01) | 0.87(0.74,1.02) | 0.69(0.50,0.92) | 0.69(0.51,0.94) | 0.71 (0.53,0.97) |
| Q3 | 0.75(0.64,0.87) | 0.72(0.60,0.86) | 0.74(0.62,0.89) | 0.82(0.61,1.10) | 0.80(0.59,1.08) | 0.83 (0.62,1.11) |
| Q4 | 0.53(0.45,0.62) | 0.49(0.40,0.62) | 0.52(0.41,0.66) | 0.67(0.50,0.92) | 0.64(0.43,0.95) | 0.70 (0.47,1.04) |
| P for trend | <0.001 | <0.001 | <0.001 | 0.040 | 0.060 | 0.140 |
| Sensitivity analysis 3 (Missing values interpolated with random forest interpolation methods) | | | | | | |
| Q1 | ref | ref | ref | ref | ref | ref |
| Q2 | 0.89(0.77,1.02) | 0.84(0.72,0.99) | 0.87(0.74,1.02) | 0.76(0.57,1.02) | 0.74(0.54,1.01) | 0.77(0.57,1.05) |
| Q3 | 0.74(0.64,0.86) | 0.68(0.56,0.81) | 0.71 (0.59,0.85) | 0.85(0.64,1.14) | 0.81(0.60,1.10) | 0.85(0.63,1.14) |
| Q4 | 0.54(0.46,0.63) | 0.47(0.38,0.58) | 0.50 (0.40,0.63) | 0.69(0.51,0.94) | 0.64(0.42,0.96) | 0.70(0.46,1.06) |
| P for trend | <0.001 | <0.001 | <0.001 | 0.046 | 0.058 | 0.142 |
| Sensitivity analysis 4 (Survey cycle adjusted for) | | | | | | |
| Q1 | ref | ref | ref | ref | ref | ref |
| Q2 | 0.88(0.77,1.01) | 0.84(0.72,0.98) | 0.85(0.72,0.99) | 0.70(0.50,0.92) | 0.68(0.49,0.94) | 0.70 (0.51,0.97) |
| Q3 | 0.75(0.64,0.87) | 0.69(0.57,0.83) | 0.70(0.58,0.85) | 0.82(0.61,1.11) | 0.79(0.58,1.08) | 0.81 (0.60,1.10) |
| Q4 | 0.53(0.45,0.62) | 0.46(0.37,0.57) | 0.48(0.38,0.61) | 0.68(0.50,0.92) | 0.63(0.41,0.96) | 0.68 (0.45,1.04) |
| P for trend | <0.001 | <0.001 | <0.001 | 0.040 | 0.063 | 0.121 |
| Sensitivity analysis 5 (Excluded participants with hypertension, CVD, diabetes and CKD) | | | | | | |
| Q1 | ref | ref | ref | ref | ref | ref |
| Q2 | 0.70(0.52,0.93) | 0.62(0.46,0.84) | 0.62 (0.46,0.84) | 1.08(0.33,3.49) | 0.84(0.26,2.72) | 0.84 (0.26,2.72) |
| Q3 | 0.56(0.43,0.74) | 0.45(0.33,0.62) | 0.45 (0.33,0.62) | 0.64(0.24,1.72) | 0.41(0.16,1.05) | 0.41 (0.16,1.05) |
| Q4 | 0.44(0.33,0.57) | 0.31(0.21,0.45) | 0.31 (0.21,0.45) | 0.72(0.24,2.20) | 0.37(0.10,1.38) | 0.37 (0.10,1.38) |
| P for trend | <0.001 | <0.001 | <0.001 | 0.386 | 0.062 | 0.062 |

HUA, hyperuricemia; OBS, oxidative balance score.

Model 1 was adjusted for demographic data (sex, age group, education level, income level, marital status).

Model 2 was adjusted for demographics data, total energy intake and HEI-2015

Model 3 was adjusted for demographics data, total energy intake, HEI-2015 and disease conditions (hypertension, CVD, diabetes and CKD).
